# Lipoprotein(a)/CD36 Interaction Drives IL-6/RhoA-GTP Signaling and miRNA Epigenetic Regulation in Coronary Artery Spasm

**DOI:** 10.3390/ph18091384

**Published:** 2025-09-16

**Authors:** Yen-Kuang Lin, Tsung-Han Hsieh, Chi-Tai Yeh, Vijesh Kumar Yadav, Iat-Hang Fong, Kuang-Tai Kuo, Nicholas G. Kounis, Patrick Hu, Ming-Yow Hung

**Affiliations:** 1 Graduate Institute of Athletics and Coaching Science, National Taiwan Sport University, No. 250 Wenhua 1st Rd., Guishan, Taoyuan 33301, Taiwan; robbinlin@ntsu.edu.tw; 2 Biostatistics Research Center, Taipei Medical University, Taipei City 11031, Taiwan; 3 Center for Management and Development, Taipei Medical University, Taipei City 11031, Taiwan; thhsieh@tmu.edu.tw; 4 Department of Medical Research and Education, Shuang Ho Hospital, Taipei Medical University, New Taipei City 23561, Taiwan; ctyeh@s.tmu.edu.tw (C.-T.Y.); 20604@s.tmu.edu.tw (V.K.Y.); 18149@s.tmu.edu.tw (I.-H.F.); 5 Continuing Education Program of Food Biotechnology Applications, College of Science and Engineering, National Taitung University, Taitung 95092, Taiwan; 6 Division of Thoracic Surgery, Department of Surgery, Shuang Ho Hospital, Taipei Medical University, New Taipei City 23561, Taiwan; ktkuo@tmu.edu.tw; 7 Department of Cardiology, University of Patras Medical School, Rion, 26504 Patras, Achaia, Greece; ngkounis@otenet.gr; 8 Department of Internal Medicine, School of Medicine, University of California, Riverside, Riverside, CA 92521, USA; dr.hu.md@gmail.com; 9 Department of Cardiology, Riverside Medical Clinic, Riverside, CA 92506, USA; 10 Division of Cardiology, Department of Internal Medicine, School of Medicine, College of Medicine, Taipei Medical University, Taipei City 11031, Taiwan; 11 Division of Cardiology, Department of Internal Medicine, Shuang Ho Hospital, Taipei Medical University, New Taipei City 23561, Taiwan; 12 Taipei Heart Institute, Taipei Medical University, Taipei City 11031, Taiwan

**Keywords:** biomarker, coronary artery spasm, cluster of differentiation 36, lipoprotein(a), molecular docking, RAS homolog family member A, RhoA GTPase, Rho-kinase, soluble CD36

## Abstract

**Background:** Lipoprotein(a) [Lp(a)]-induced inflammation contributes to coronary artery spasm (CAS) by the contraction of vascular smooth muscle cells. However, the interaction between Lp(a) and soluble CD36 (sCD36)/interleukin (IL)-6/RAS Homolog Family Member A (RhoA)-GTP signaling pathway has not been evaluated. **Methods:** We investigated the relevance of Lp(a)/CD36 signaling in CAS patient monocyte-derived macrophages (PMDMs) and a human coronary artery smooth muscle cell (HCASMC) line using expression profile correlation analyses, molecular docking, RNA sequencing, flow cytometry, immunoblotting, and quantitative reverse transcription polymerase chain reaction. **Results:** Plasma Lp(a) and sCD36 levels in 41 CAS patients were significantly higher (*p* = 0.001) and positively correlated (r^2^ = 0.3145, *p* < 0.001), a trend not observed in 36 non-CAS controls. RNA sequencing indicated a significant co-overexpression of CD36 and RhoA in Lp(a)-treated CAS PMDMs and HCASMCs, of which the mRNA and protein expression of CD36 and RhoA were significantly enhanced (*p* < 0.001) dose-dependently. Lp(a) rather than LDL preferentially induced CD80+ PMDM (M1) polarization. In HCASMCs, the CD36 knockdown using either short hairpin RNA or natural biflavonoid amentoflavone suppressed Lp(a)-upregulated protein expression of CD36, RhoA-GTP, IL-6, tumor necrosis factor (TNF)-α, nuclear factor (NF)-κB, and CD80; however, overexpressed CD36 increased their levels. Lp(a) decreased and amentoflavone increased the epigenetic expression of CD36 inhibitors, miR-335-5p, and miR-448, respectively. Reciprocally, an miRNA inhibitor or mimic could magnify or diminish Lp(a)-induced CD36, TNF-α, NF-κB and IL-6 expressions in HCASMCs, respectively. **Conclusions:** Elevated Lp(a) levels upregulate the CD36-dependent TNF-α/NF-κB/IL-6/RhoA-GTP signaling pathway in CAS PMDMs and HCASMCs, indicating that Lp(a)/CD36 inflammatory signaling, HCASMC activation, and macrophage M1 polarization mediate CAS development.

## 1. Introduction

Rest- and/or effort-associated coronary artery spasm (CAS), an excessive vasoconstriction of vascular smooth muscle cells (VSMCs) resulting in total or subtotal vessel obstruction, plays a key role in acute coronary syndrome (ACS), such as unstable angina, ischemia, myocardial infarction without obstructive coronary artery disease, and sudden cardiac death [1,2]. CAS has been linked to inflammation marked by elevated peripheral monocyte count [3], levels of C-reactive protein (CRP) [4], and interleukin (IL)-6 [5]; among them, the increased IL-6 and CRP levels reduce endothelial nitric oxide production [6,7], leading to CAS development. Among lipoproteins, lipoprotein(a) [Lp(a)] is a risk factor for CAS [8]. The key proinflammatory oxidized phospholipids are predominantly found on apolipoprotein(a) isoforms of Lp(a), with only small amounts on low-density lipoprotein (LDL) and high-density lipoprotein [9]. Moreover, in vitro transfer studies show that oxidized LDL preferentially donates oxidized phospholipids to Lp[a], as opposed to LDL, in a time- and temperature-dependent manner [10]. We previously demonstrated that Lp(a)-triggered inflammation mediated CAS through α7-nicotinic acetylcholine receptor (α7-nAChR)/p38 MAPK/IL-6/RAS Homolog Family Member A (RhoA)-GTP signaling induction, CAS patient monocyte-derived macrophages (PMDMs) M1 polarization, and activation of human coronary artery smooth muscle cells (HCASMCs) [11]. In addition, the uncontrolled VSMC contraction in CAS is related to the RhoA GTPase/Rho-kinase (ROCK1, ROCK2) pathway, which further induces inflammation and oxidative stress [12]. Although these observations highlight the important role of Lp(a) in modulating the inflammation-associated development of CAS, and while scavenger receptors’ recognition of oxidized lipoproteins on phagocytic cells evolve with the innate immune system, the interactions between Lp(a) and scavenger receptors have not been evaluated in CAS.

The scavenger receptor cluster of differentiation 36 (CD36), a class B protein (SR-B2) with dual functions as both an oxidized LDL receptor and a fatty acid transporter in a tissue-specific manner [13], is widely expressed in many immune and non-immune cells [14]. While CD36 is preferentially expressed in tissues performing very active fatty acid metabolism, such as the heart, the binding of modified lipoproteins to CD36 might regulate CAS development [14]. CD36 promotes inflammation and has been associated with ACS, among which the CD36 gene expression is ≥1.91-fold in the clinical spectrum of ACS in comparison with a non-ACS control group [15]; however, whether circulating soluble CD36 (sCD36) is connected with CAS development is unknown [15]. Notably, the activation of CD36 by hexarelin, a growth hormone-releasing peptide, induces a dose-dependent increase in coronary perfusion pressure and CAS in isolated perfused hearts of rats and mice [16], suggesting CD36 may mediate CAS evolution. Nonetheless, scarce data are available on Lp(a) in relation to CD36 in CAS. On the other hand, smoking and CRP are two important risk factors for CAS [17] and are strongly correlated with insulin resistance [18]. Moreover, considering the significance of CD36 in prediabetes [19], the redistribution of CD36 in cardiomyocytes from intracellular compartments to the sarcolemma is one of the earliest changes in insulin resistance [20], which has a significant positive association with CAS [21]. Reciprocally, CAS is a risk factor for incident diabetes regardless of sex [22]. In addition, in glucose-intolerant subjects, sCD36 is positively associated with insulin resistance, IL-6, fasting triglycerides, and platelet count [23]. In early molecular events of ischemic brain injury, CD36 leads to nuclear factor-κB (NF-κB) activation and postischemic inflammation [24]. Moreover, RhoGTPases play a context-dependent positive or negative regulatory role in NF-κB activation, as the RhoA–NF-κB interaction has been shown to be important in tumor necrosis factor-α (TNF-α)-activated NF-κB processes [25]. NF-κB can activate the myosin light chain kinase/myosin light chain-2 pathway and upregulate endothelin-1 expression by binding to their promoter regions, thus inducing VSMC contraction in an in vitro model of CAS [26]. Notably, Lp(a) inhibits lipopolysaccharide-induced TNF-α production by human mononuclear cells [27]. Collectively, these studies indicate that the effects of CD36 as well as sCD36 on the TNF-α/NF-κB/IL-6/RhoA-GTP signaling pathway may mediate CAS development.

**Figure 1:**
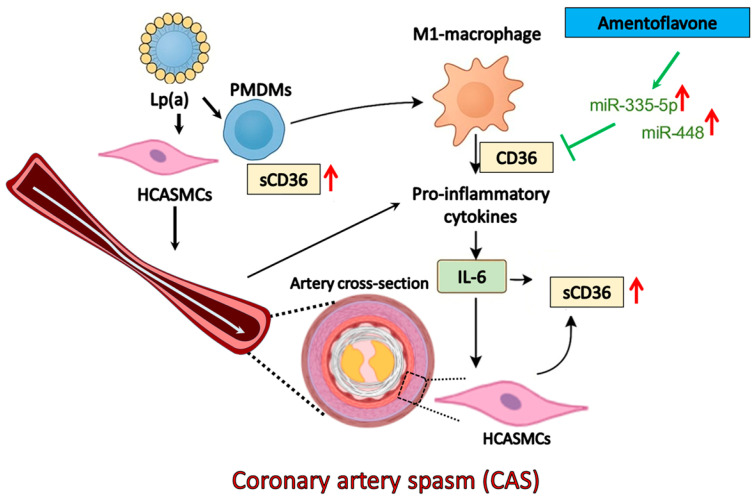
Schematic illustration of the proposed mechanism linking Lp(a), CD36, and RhoA/ROCK signaling in coronary artery spasm (CAS) pathogenesis. Lp(a) interacts with circulating monocytes, leading to CD36 upregulation and binding of oxidized phospholipids. This promotes proinflammatory cytokine production and LxR activation, which subsequently triggers RhoA/ROCK pathway activation in vascular smooth muscle cells, enhancing vasoconstriction and CAS development. The upward arrow symbolizes an increase. The black arrow head of solid lines means activating cellular functions. The green arrow and solid line denote activating and inhibitory epigenetic regulation, respectively.

Clinically, CAS-induced repeat transient myocardial ischemia–reperfusion can stimulate proinflammatory responses from macrophages and coronary VSMCs [11,28], which can reciprocally activate CAS [29]. As a result, the dysregulated immunometabolism [30] may have a crucial role in the occurrence of CAS. Recent studies indicate that the dual functions of CD36 can influence innate and adaptive immune cell activation and differentiation fates [31]. In monocytes, CD36 is upregulated by peroxisome proliferator-activated receptor gamma and cytokines, such as macrophage colony-stimulating factor, IL-4, and IL-10, which are involved in differentiation into dendritic cells and reparative M2 phenotypes [32,33]. In contrast, lipopolysaccharide and dexamethasone downregulate CD36 expression [34]. However, whether Lp(a) up- or downregulates CD36 expression in CAS remains unclear. On the other hand, the regulation of CD36 expression at both the transcriptional and posttranslational levels varies among different cell types, differentiation states, as well as exposure to soluble mediators [31]. Under chronic inflammation, oxidant stress, hyperlipidemia, or diabetes, CD36 deficiency alleviates atherosclerosis and thrombosis to maintain homeostasis [35]. In addition, microRNAs (miRNAs), small non-coding RNAs as key regulators in various inflammatory diseases, have gained attention in the past two decades for their potential to serve as biomarkers and therapeutic targets. In the diabetic heart, for example, miR-320 and CD36 reinforce each other in a positive feedback loop that perpetuates lipotoxicity and diastolic dysfunction [36]. As a result, whether LP(a)/CD36 interactions in CAS have a distinctive cardiac target signature through which complex disease-related molecular changes can be reprogrammed is important for a concerted therapeutic effect. We therefore analyzed (1) the Lp(a)-associated mRNA and protein expression levels of CD36 in the PMDMs of CAS patients and HCASMCs; (2) the relationship of CD36 and monocyte-to-macrophage differentiation and polarization based on CD80 or CD206 positivity in CAS PMDMs; (3) the probable positive interaction and modulatory loop between Lp(a), CD36, and the TNF-α/NF-κB/IL-6/RhoA-GTP signaling pathway in HCASMCs; (4) the role of CD36-dependent epigenetic regulation of anti-inflammatory microRNAs (miRNAs) expression in HCASMCs (Figure 1).

## 2. Results

### 2.1. Study Cohort Baseline Characteristics

A total of 77 patients were enrolled in the study (median age = 59.0 years; interquartile range, 48–66 years). The CAS group compared with the control group were more likely to be male, current smokers (*p* < 0.05), had significantly lower high-density lipoprotein, higher sCD36 and Lp(a) levels (*p* = 0.001, respectively), and had significantly higher monocyte and macrophage counts and hemoglobin values (all *p* < 0.05) (Table 1). In CAS patients, single-vessel spasm was most common and usually occurred in the right coronary artery. There were no significant differences in medication usage across the groups prior to undergoing coronary angiography. Following coronary angiography, the proportion of patients receiving beta blockers was notably lower in the CAS group compared with the control group, while the use of calcium channel blockers was appreciably higher in the CAS group (Table 1).

### 2.2. Correlation of Lp(a) with sCD36

**Figure 2:**
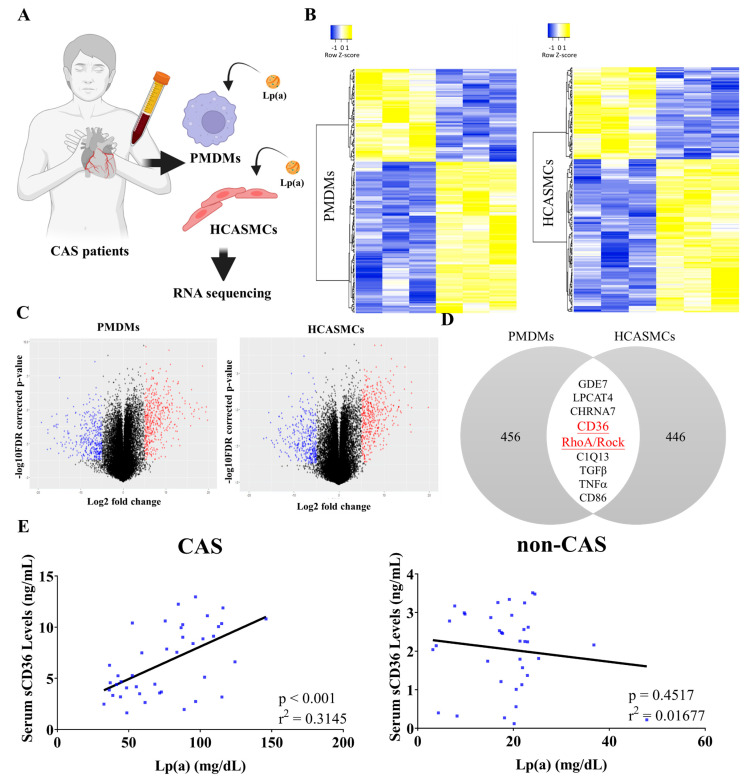
Lipoprotein(a) (Lp(a)) upregulated CD36 and RhoA expression, which was positively correlated with soluble cluster of differentiation 36 (sCD36) levels in coronary artery spasm (CAS). (**A**) RNA sequencing after isolation and treatment of CAS patient monocyte-derived macrophages (PMDMs) and a human coronary artery smooth muscle cell (HCASMC) line. (**B**) Heatmap of differentially expressed genes (DEGs) from CAS PMDMs and HCASMCs. The colored codes from blue to yellow indicate expression levels from low (in blue) to high (in yellow). (**C**) Volcano plot of the expression levels of the PMDMs and HCASMCs compared with those of the control groups. The blue and red dots indicate the lowest level and the highest level of expression, respectively. (**D**) Upregulated CD36 and RAS Homolog Family Member A (RhoA)-GTP/Rho-kinase (ROCK) DEGs (at the center) from PMDMs and HCASMCs are identified in the Venn diagram. Only the red-labeled genes (CD36 and RhoA/ROCK) meet the adjusted significance threshold (False Discovery Rate < 0.01), while the other overlapping genes shown in black are less statistically significant (False Discovery Rate < 0.05). (**E**) Lp(a) levels were positively correlated with sCD36 in CAS patients but not in non-CAS controls.

The microarray sequencing of Lp(a)-stimulated PMDMs of CAS patients (n = 3) and HCASMCs (n = 3) were analyzed to examine the expression correlation between them (Figure 2A). In the differential expression genes (DEGs) analysis under Lp(a) treatment, distinct upregulated or downregulated patterns of gene expression between CAS PMDMs and HCASMCs were observed and based on the cut-off criteria log2FC > 1.5 and *p* < 0.05, including a total of 1955 DEGs consisting of 749 upregulated and 1206 downregulated DEGs from stimulated CAS PMDMs, and a total of 1955 DEGs consisting of 749 upregulated and 1206 downregulated DEGs from HCASMCs cells, as shown in the heatmap displaying sample clusters (Figure 2B) and the volcano plot (Figure 2C). The Venn diagram, which displays the differences in DEGs between Lp(a)-treated CAS PMDMs and HCASMCs, demonstrates that CD36 and RhoA genes were among the overlapping and overexpressed DEGs (Figure 2D). This comparison identified the genes with expression changes affected by Lp(a) in CAS PMDMs and HCASMCs. Furthermore, Lp(a) levels were positively correlated with sCD36 levels in CAS (r^2^ = 0.3145, *p* < 0.001) but not in non-CAS patients (Figure 2E).

### 2.3. Lp(a) Stimulated CD36 and RhoA Expression in PMDMs, HCASMCs, and Preferentially Induced PMDM M1 Polarization

**Figure 3:**
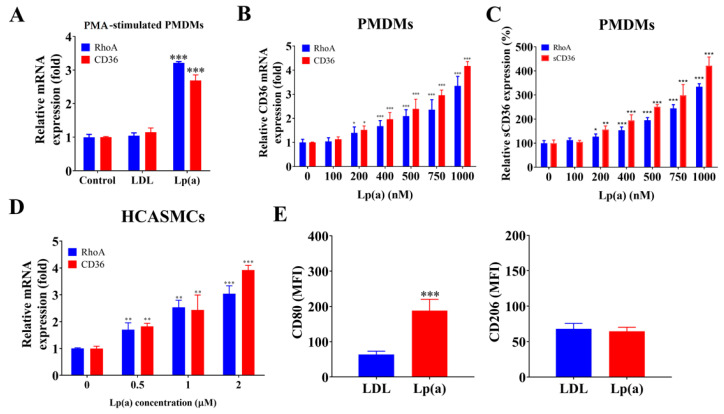
Lp(a)-induced CD36 and RhoA expression in CAS PMDMs and HCASMCs as well as M1 polarization. (**A**) Graphical representation of the effect of 500 nM low-density lipoprotein (LDL) or Lp(a) on the relative expression of RhoA or CD36 in the CAS PMDMs. (**B**,**C**) Histograms showing the dose-dependent effect of 100 nM–1000 nM Lp(a) on the relative expression of CD36/RhoA mRNA or relative luciferase reporter activity of sCD36/RhoA in the CAS PMDMs. (**D**) Investigation of 0.5 nM–2 nM Lp(a) for the dose-dependent relative expression of CD36/RhoA mRNA in HCASMCs. (**E**) Flow cytometry cell count histograms depicting the effect of 500 nM LDL or Lp(a) treatment for 24 h on the CD80 or CD206 median fluorescence intensity of CAS PMDMs, in which colors are blue for control antibody and red for target antibody. * *p* < 0.05, ** *p* < 0.01, *** *p* < 0.001. MFI: mean fluorescence intensity.

Commercially available native human Lp(a) was used in all functional assays. When PMDMs were stimulated with phorbol-12-myristate-13-acetate (PMA) in the absence (control) or the presence of 500 nM LDL or Lp(a), Lp(a) treatment resulted in a 2.5-fold increase in CD36 mRNA and a 3.5-fold increase in RhoA mRNA compared with both the untreated control and LDL-treated groups (Figure 3A). In parallel assays, exposure to 100 nM–1 μM, corresponding to 2.5–25 mg/dL, Lp(a) dose-dependently increased (*p* < 0.001) CD36 and RhoA mRNA, as well as sCD36 and RhoA protein expression levels (Figure 3B,C). A dose-dependent induction of CD36 and RhoA mRNA expression was also observed in HCASMCs incubated with 0.5 to 2 μM, Lp(a) for 48 h (Figure 3D). Our CAS PMDMs were not polarized before Lp(a) exposure. Cells were differentiated from peripheral monocytes under phorbol-12–myristate-13–acetate stimulation and then directly treated with Lp(a) to maintain an unpolarized baseline state, allowing us to assess the intrinsic effect of Lp(a) on macrophage polarization dynamics. In CAS PMDMs, 500 nM Lp(a) induced a 3.2-fold greater increase in CD80+ macrophage fluorescence intensity than 500 nM LDL (*p* < 0.001) (Figure 3E, left). Neither LDL nor Lp(a) exposure produced a significant change in the mean fluorescence intensity of the CD206+ macrophage population (Figure 3E, right), indicating that Lp(a) specifically promotes M1 macrophage polarization in patients with CAS. To further validate this shift in macrophage phenotype, qRT-PCR analysis of canonical M1/M2 markers was performed in CAS PMDMs following 1 μM Lp(a) exposure for 24 h (Supplementary Figure S1). Lp(a) significantly increased inducible nitric oxide synthase and interleukin-12 mRNA expression (M1 markers) while reducing arginase-1 and interleukin-10 expression (M2 markers). These transcriptional changes were consistent with a robust shift toward a proinflammatory M1 phenotype, in line with the flow cytometry findings.

### 2.4. In Silico Molecular Docking to Examine Lp(a)/sCD36 Binding Interactions

**Figure 4:**
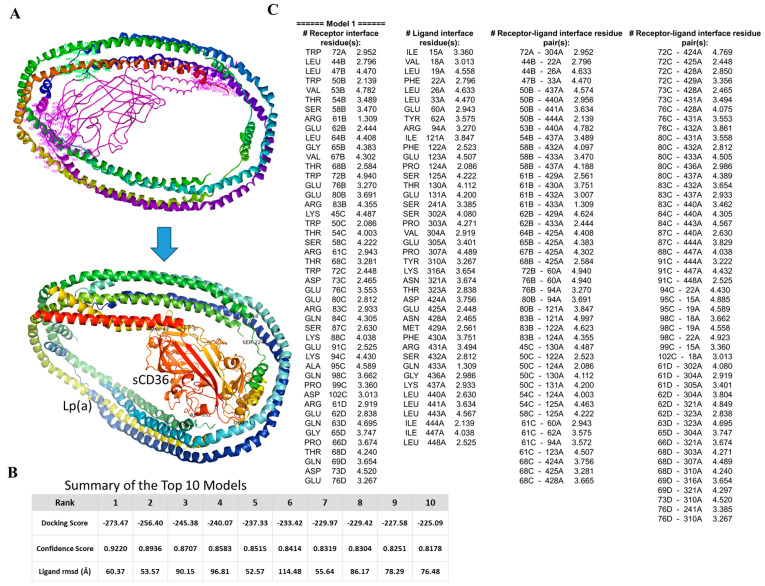
Docking and interface analysis of apolipoprotein(a) on Lp(a) with the sCD36 ectodomain. (**A**) Best-ranked docked complex: rainbow-colored apolipoprotein(a) bonds to the Asp238–Glu242 acidic patch of sCD36 (orange), blocking the lipid tunnel. (**B**) Score table for the 10 top models showed a leading docking score of −273.47 kcal mol^−1^ with all poses clustering within ≤1.7 Å ligand Root-Mean-Square Deviation. (**C**) Interface map highlighting the dominant Lys70/Lys74↔Asp238/Glu240 salt bridges that underlay the favorable −122 kcal mol^−1^ HADDOCK score and the 2.6 Å backbone Root-Mean-Square Deviation maintained during 100 ns molecular dynamics.

By using AlphaFold models of the CD36 ectodomain (AF-Q07954-F1, residues 30–439) and apolipoprotein(a) on Lp(a), we ran a two-step workflow: 70,000 rigid-body poses were generated with ClusPro, and the 30 best clusters were flexibly refined in HADDOCK (online server: http://hdock.phys.hust.edu.cn/, accessed on 12 June 2025). Cartoon view of the best-ranked complex is shown in Figure 4A. The top ClusPro solution scored −273.47 kcal mol^−1^ (confidence 0.922), and all 10 highest poses laid within −225 to −273 kcal mol^−1^ and ≤1.7 Å ligand Root-Mean-Square Deviation (Figure 4B). HADDOCK water refinement kept 128 near-identical poses averaging a score of −122 kcal mol^−1^, which was about 20 kcal mol^−1^ better than the next cluster and placed the lysine-rich groove of apolipoprotein(a) squarely on the Asp238–Glu242 acidic patch of CD36, burying ~1.6 × 10^3^ Å^2^ of surface through a tight Lys70/Lys74↔Asp238/Glu240 salt-bridge network (Figure 4C). A 100 ns explicit-solvent molecular dynamics simulation (ff19SB/OPC) confirmed stability, in which the complex Root-Mean-Square Deviation rose to only 2.6 ± 0.2 Å after equilibration, the interface held ~12 H-bonds and 5 salt bridges, and Molecular Mechanics Generalized Born Surface Area binding energy settled at −54 ± 5 kcal mol^−1^. Altogether, these data support a single, high-affinity pose in which Lp(a) clamped onto sCD36 through a lysine-acid “Velcro” interface to drive the conformational changes linked to outside-in signaling, offering a structural rationale for Lp(a)-induced CD36-dependent macrophage activation and providing clear targets for cross-link validation.

### 2.5. CD36 Knockdown Reduced Lp(a)-Induced Proinflammatory Signaling in HCASMCs

**Figure 5:**
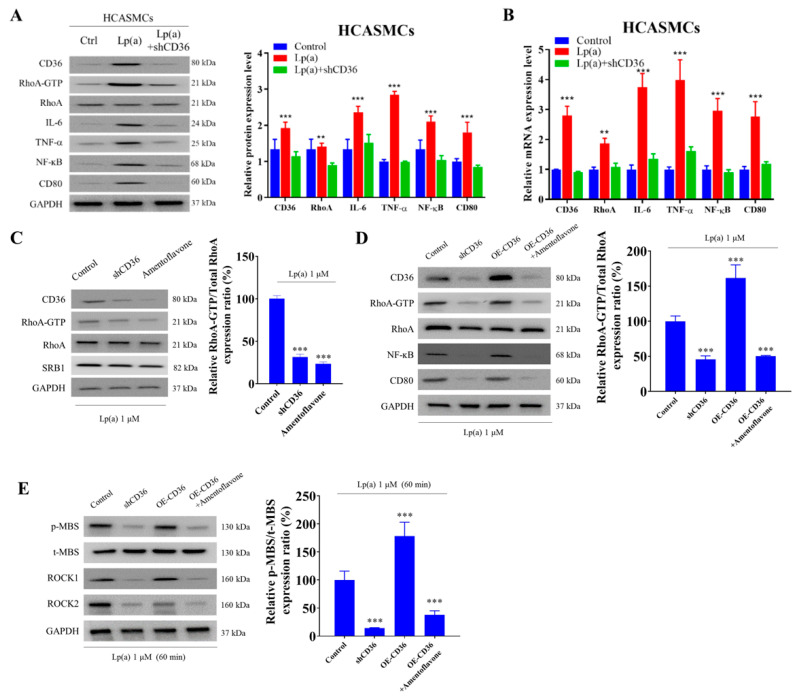
The impact of short hairpin RNA for CD36 (shCD36), CD36 overexpression (OE-CD36) vector, and amentoflavone on Lp(a)-induced cellular signaling pathways in HCASMCs. (**A**,**B**) Immunoblotting and quantitative reverse transcription polymerase chain reaction investigating the effects of Lp(a) alone or with shCD36 knockdown demonstrated that a reduction in fold change in CD36 mRNA and protein expression levels could significantly downregulate tumor necrosis factor (TNF)-α/nuclear factor kappa B (NF-κB)/interleukin (IL)-6/RhoA-GTP signaling pathways. (**C**) In Lp(a)-treated HCASMCs, shCD36 and amentoflavone, in contrast with control, significantly reduced the RhoA-GTP/RhoA ratio using a pull-down assay with the glutathione-S-transferase-Rhotekin-Rho-binding domain. (**D**) While HCASMCs transfected with OE-CD36 vector showed a significantly higher RhoA-GTP/RhoA ratio than CD36 knockdown cells and the vehicle vector control, the addition of amentoflavone to the OE-CD36 medium significantly decreased the CD36 expression level similar to that of shCD36. (**E**) Representative Western blot photo images showing that in 1 μM Lp(a)-treated HCASMCs, the addition of shCD36, OE-CD36, or OE-CD36+amentoflavone for 60 min, compared with controls, significantly decreased, increased, or decreased ROCK activity, respectively. The ROCK activity was expressed as a relative blot density ratio, which was the expression ratio of phosphorylation level of myosin-binding subunit of myosin light chain phosphatase (pMBS) sample density/total MBS (tMBS) sample density. ** *p* < 0.01, *** *p* < 0.001; GAPDH served as loading control.

The short hairpin RNA for CD36 (shCD36) knockdown remarkably suppressed Lp(a)-induced CD36 protein expression in HCASMCs by 81% and significantly decreased both protein and mRNA expression levels of RhoA-GTP, RhoA, IL-6, TNF-α, NF-κB, and CD80 (Figure 5A,B). In Lp(a)-treated HCASMCs, shCD36 and a natural biflavone CD36 antagonist amentoflavone significantly reduced the RhoA-GTP/RhoA ratio using a pull-down assay with the glutathione-S-transferase-Rhotekin-Rho-binding domain (Figure 5C). On the other hand, while HCASMCs transfected with CD36 overexpression (OE-CD36) vector, cDNA-CD36, showed a significantly higher RhoA-GTP/RhoA ratio than CD36 knockout cells and the vehicle vector control, the addition of amentoflavone to OE-CD36 medium significantly decreased the CD36 expression to the level similar to that observed with shCD36 (Figure 5D). In 1 μM Lp(a)-treated HCASMCs, the addition of shCD36, OE-CD36, or OE-CD36+amentoflavone for 60 min, compared with controls, significantly decreased, increased, or decreased ROCK activity, which was the downstream effector of Rho GTPase and RhoA-GTP, respectively (Figure 5E). Our experimental model demonstrated that SR-B1 expression remained unchanged following Lp(a) stimulation, CD36 knockdown, or amentoflavone treatment, thereby providing additional evidence that CD36 is the primary receptor responsible for mediating Lp(a)-induced signaling in CAS.

### 2.6. Epigenetic Regulation of Lp(a)-Triggered CD36, IL-6, TNF-α, and NF-κB Expression in HCASMCs

**Figure 6:**
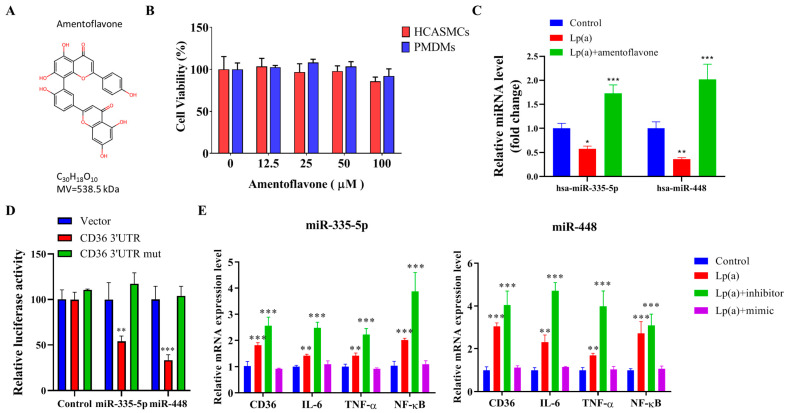
Amentoflavone exerted epigenetic regulation of CD36, IL-6, TNF-α, and NF-κB mRNA expression through miR-335-5p and miR-448, which mediated the Lp(a)/CD36 interaction in HCASMCs. (**A**) Amentoflavone has a molecular formula of C_30_H_18_O_10_ and molar mass of 538.45 g/mol. (**B**) Graphical representation of the effect of 12.5 μM–100 μM amentoflavone on the viability of HCASMCs and CAS PMDMs. (**C**) Exposure to 10 μM amentoflavone significantly reversed the 1 μM Lp(a)-induced downregulation of hsa-miR-335-5p and hsa-miR-448 expression levels in HCASMCs, which were also significantly higher than non-Lp(a)-treated controls. (**D**) In HCASMCs, dual luciferase reporter gene assay demonstrated that the targeting sequence for miR-335-5p and miR-448 was identified in the 3′-UTR of CD36 mRNA using constructs containing the predicted targeting sequence (CD36 3′-UTR) and mutated targeting sequence (CD36 3′-UTR mut) cloned into the 3′-UTR of the reporter gene. (**E**) In HCASMCs, the miR-335-5p’s and miR-448’s inhibitors largely increased, and mimics decreased, the 1 μM Lp(a)-induced target mRNA translation, respectively, such as CD36, IL-6, TNF-α, and NF-κB. * *p* < 0.05, ** *p* < 0.01, *** *p* < 0.001.

Amentoflavone has a molecular formula of C_30_H_18_O_10_ (Figure 6A). Treatment with 12.5 μM–100 μM amentoflavone did not affect cell viability in CAS PMDMs and HCASMCs (Figure 6B). In HCASMCs, exposure to 10 μM amentoflavone significantly reversed the 1 μM Lp(a)-induced downregulation of hsa-miR-335-5p and hsa-miR-448 expression levels to the extent that they were also significantly higher than those of non-Lp(a)-treated controls (Figure 6C). Consistent with these findings, analysis using miRTarBase and MirTarget identified multiple CD36-targeting miRNAs, visualized as a radial connectivity chart linking CD36 with candidate miRNAs (Supplementary Figure S2A). Notably, amentoflavone upregulated miR-335-5p and miR-448 while concurrently downregulating miR-455-5p and miR-155-5p (Supplementary Figure S2B). In HCASMCs, a dual luciferase reporter gene assay demonstrated that the targeting sequences for miR-335-5p and miR-448 were identified in the 3′-UTR of CD36 mRNA (Figure 6D). Furthermore, in HCASMCs, inhibition of miR-335-5p or miR-448 markedly increased Lp(a)-induced target mRNA translation of CD36, IL-6, TNF-α, and NF-κB, whereas their mimics suppressed this response (Figure 6E), supporting the role of these miRNAs as key mediators of the Lp(a)/CD36 interaction.

## 3. Discussion

We demonstrated that, compared with controls, CAS patients had higher serum Lp(a) levels, which were positively correlated with sCD36 levels in CAS but not in controls. Lp(a) preferentially induced PMDM M1 polarization in CAS and increased the expression of RhoA and CD36 in CAS PMDMs and HCASMCs and the subsequent elevated sCD36 protein expression in PMDMs. Our molecular docking predicted a good binding mode and affinity of sCD36 within Lp(a) binding sites in a stable and favorable manner. In Lp(a)-treated HCASMCs, shCD36 significantly decreased both protein and mRNA expression levels of CD36, RhoA-GTP, RhoA, IL-6, TNF-α, NF-κB, and CD80. In addition, shCD36 and amentoflavone significantly reduced the RhoA-GTP/RhoA ratio. On the other hand, while Lp(a)-treated HCASMCs showed a significantly higher RhoA-GTP/RhoA ratio in cells transfected with OE-CD36 than shCD36, the addition of amentoflavone to the OE-CD36 medium significantly downregulated CD36 expression to a level similar to that of shCD36. Amentoflavone exerted epigenetic regulation of CD36, IL-6, TNF-α, and NF-κB mRNA expression through miR-335-5p and miR-448, which mediated the Lp(a)/CD36 interaction in HCASMCs. Our novel pathways of Lp(a)-triggered CD36-dependent TNF-α/NF-κB/IL-6/RhoA-GTP signaling induction, macrophage M1 polarization, and HCASMC activation may be one of the important mechanisms contributing to CAS development. While our present work was designed to dissect the specific role of the Lp(a)/CD36 axis in driving RhoA/ROCK-dependent inflammatory signaling in CAS, we acknowledge that vascular smooth muscle hyperreactivity is a multifactorial process likely influenced by additional pathways. The emerging literature suggests that scavenger receptor-independent mechanisms—such as endothelin-1 signaling, oxidative stress-driven MAPK activation, TLR2/4-mediated activation, and purinergic receptor pathways—may operate in parallel or compensate when CD36 activity is modulated [37,38,39,40].

Lp(a) is a well-established genetic risk factor for coronary artery disease (CAD), while its pathogenic mechanism in CAS has remained unclear [41]. Despite that oxidized LDL represents a biologically relevant comparator, as oxidized phospholipids are proinflammatory, our choice of native LDL in the present study was based on the consideration to contrast Lp(a) with a non-oxidized lipoprotein of similar density to isolate the contribution of the apolipoprotein(a)-bound oxidized phospholipid Lp(a). However, we acknowledge that prior studies have shown oxidized LDL to be a potent inducer of CD36 expression and downstream inflammatory signaling [42], and that oxidized LDL preferentially donates oxidized phospholipids to Lp(a), as opposed to LDL [10]. In our post hoc stratified analyses, the positive correlation between Lp(a) and sCD36 levels, as well as the Lp(a)-associated upregulation of CD36 and RhoA expression in PMDMs and HCASMCs, remained significant in both smokers and non-smokers (all *p* < 0.05), without significant interaction effects between smoking status and Lp(a) levels. In previous studies, smoking had shown no effect on Lp(a) levels [43], and no significant interactions were observed between Lp(a) and smoking [44]. Altogether, our observed associations are independent of smoking-related influences. Nonetheless, Lp(a) can be upregulated in chronic inflammatory disorders, such as rheumatoid arthritis or Crohn’s disease, and among patients receiving hemodialysis, further implicating its role in immune-mediated cardiovascular risk [45,46,47]. Lp(a) levels also increase under acute stress conditions such as surgery or myocardial infarction, highlighting its involvement in innate immunity [48]. Lp(a) is found only in monkeys, apes, and humans [49], and some species lack kringle V. Human apolipoprotein(a) kringles are specialized for ligand interactions [50], particularly with lysine-containing substrates, due to both kringle V and a functional lysine binding site in kringle IV type 10. Although its exact receptor remains unknown, Lp(a) interacts with several receptors, including scavenger receptors like CD36 [51]; however, the roles of these receptors, including lipoprotein receptors, toll-like and scavenger receptors, lectins, and plasminogen receptors, remain unclear [51]. Because the absence of Lp(a) in most experimental animals poses limitations for modeling Lp(a)/CD36 interactions directly, we recommend a multi-tiered preclinical validation approach. First, in vitro assays using primary HCASMCs and PMDMs from CAS patients can provide direct mechanistic evidence. Second, transgenic mouse models expressing human apolipoprotein(a), which has already been used in Lp(a) atherogenesis studies [52], can be leveraged to examine vascular reactivity and inflammation in vivo. Third, applications of CRISPR/Cas9 technology in a CD36 humanized mouse model [53], in combination with Lp(a)-transgenic backgrounds, would enable physiologically relevant testing of CD36-targeting therapeutics. Finally, non-human primate models with naturally higher Lp(a) levels may offer an additional platform for pharmacodynamic evaluation [54,55].

Our previous studies showed that Lp(a) correlates with IL-6 in CAS, contrary to the lack of such correlation in CAD [11,56], suggesting an inflammatory mechanism distinct from that of CAD. The current study built upon these findings and showed that Lp(a)-induced CD36 expression contributed to CAS pathogenesis by driving inflammation and VSMC dysfunction [57,58]. In our previous study [11], elevated IL-6 was shown to contribute to CAS development by amplifying Lp(a)-induced signaling. Nevertheless, the present study indicated that in HCASMCs, Lp(a) also triggered IL-6 production downstream of CD36 activation, suggesting a context-dependent bidirectional relationship. Notably, while systemic IL-6 in vivo may prime inflammatory pathways that enhance Lp(a) effects, in our in vitro setting, Lp(a)/CD36 engagement directly induced IL-6 expression along with TNF-α, NF-κB, and RhoA-GTP. Thus, IL-6 can act as both an upstream modulator in the whole-organism inflammatory milieu and a downstream effector of CD36 signaling in HCASMCs. While class B scavenger receptors share structural similarities, their ligand specificities and functional roles differ. SR-B1 is primarily involved in selective uptake of high-density lipoprotein cholesterol and reverse cholesterol transport, whereas CD36 (SR-B2) is preferentially expressed in metabolically active tissues such as the heart and has high affinity for oxidized phospholipid-rich lipoproteins, including Lp(a). The proinflammatory oxidized phospholipids are predominantly carried on apolipoprotein(a) isoforms of Lp(a) [9,10], a ligand profile more consistent with CD36 recognition than SR-B1. Moreover, prior mechanistic studies demonstrate that CD36 activation, but not SR-B1 activation, induces vasoconstrictive responses and coronary perfusion pressure increases [16]. In our experimental model, we confirmed that SR-B1 expression was unaffected by Lp(a) stimulation, CD36 knockdown, or amentoflavone treatment, further supporting CD36 as the principal receptor mediating Lp(a)-driven signaling in CAS. Despite CD36 being associated with lipid uptake and foam cell formation in atherosclerosis, our findings highlight a contrasting role in CAS, where it mediates proinflammatory signaling and HCASMC hyperreactivity [13,14,59,60]. Nonetheless, although CD36 deficiency is associated with reduced risks for cardiovascular diseases, the morbidity of CAD is significantly higher in CD36 deficiency patients suffering from severe atherosclerosis [61], implying that the status of CD36 deficiency might be atherogenic rather than VSMC contraction-prone. These observations imply that CD36 is a context-dependent multifunctional receptor and that both upregulation and deficiency of this protein may increase the cardiovascular risk.

Additionally, we observed that Lp(a) increased RhoA and CD36 expression in a dose-dependent manner in CAS-derived PMDMs and HCASMCs, resulting in elevated sCD36 protein expression in PMDMs. Despite limited understanding of the RhoA signaling network within the cardiovascular context, especially given its ambiguous outcomes in both in vitro and in vivo models [62], our findings align with prior observations linking RhoA/ROCK activity to NF-κB activation in intestinal inflammation [63] and increased RhoA and ROCK mRNA expression at CAS-affected coronary sites [64]. Though Lp(a) is known to activate HCASMCs via α7-nAChR/p38 MAPK/IL-6/RhoA-GTP signaling [11], the interplay between Lp(a), CD36, and RhoA in humans remains underexplored. Our data suggests a positive feedback mechanism among these three markers. Furthermore, both CD36 and RhoA mRNA/protein expression increased in a dose-dependent manner in HCASMCs when exposed to 0.5–2 μM Lp(a), with the response beginning to plateau at the upper concentration range. This apparent “threshold” likely reflected receptor–ligand binding saturation, where maximal occupancy of CD36 and engagement of downstream signaling pathways had been reached. While we did not perform formal kinetic binding analyses (e.g., Scatchard plots) in the present study, this plateau effect in the results suggests a saturable mechanism consistent with receptor-mediated signaling rather than non-specific effects. Notably, HCASMCs demonstrated increased CD80 expression following Lp(a) stimulation. Although CD80 is predominantly found on immune cells like B cells, monocytes, T cells, and dendritic cells, this upregulation in HCASMCs may point to an immunological checkpoint role during CAS development [65,66].

Data regarding human monocyte-to-macrophage differentiation and polarization upon Lp(a) exposure remain limited. However, interspecies differences in gene expression profiles during macrophage polarization have been documented [67]. Immunometabolism, which is the study of how the competing cellular aerobic and anaerobic metabolisms influence immune cell differentiation and function [68], particularly in myocardial ischemia, is emerging as a promising therapeutic concept [69]. CD36 stands out as a central regulator within this framework. Immune modulation holds promise across diverse pathological contexts [70], particularly as M1 macrophages exacerbate inflammation. In CAD, high Lp(a) levels are associated with increased CD36 expression on monocytes without corresponding changes in receptors such as CD163 and CD206 [71]. Similarly, in atherosclerosis, enhanced M1 marker expression (e.g., CD40/CD86) strengthens adaptive immunity [72]. Our findings indicate a comparable upregulation of CD80 in CAS PMDMs linked to pathogenic eicosanoid production. As IL-6 and TNF-α are hallmark cytokines of M1 polarization [73,74], it is plausible that Lp(a) fosters a proinflammatory cellular environment through CD36-mediated signaling in CAS PMDMs.

This study is the first to delineate how Lp(a)-triggered CD36-dependent monocyte/HCASMC interactions upregulate the expression of TNF-α, NF-κB, IL-6, and RhoA-GTP in VSMC dysfunction in CAS development. MiRNAs regulate gene expression by targeting mRNAs for gene silencing or translational repression [75]. Expression levels of certain miRNAs change in pathological states, including malignancies, acute kidney injury, and CAD [76]; hence, they might potentially control the inflammatory cascade in CAS. Through databases like miRTarBase and MirTarget, we identified miR-335-5p and miR-448 as novel regulators of CD36. MiR-335-5p has been identified as a possible biomarker of non-alcoholic fatty liver disease, modulating genes involved in lipid metabolism, such as peroxisome proliferator-activated receptor-γ (PPAR-γ) and sterol regulatory element-binding protein1c (SREBP1c) [77]. PPAR-γ agonists added to calcium channel blockers significantly reduced CAS compared with calcium channel blockers alone after 6 months of treatment [78]. In non-alcoholic fatty liver disease, CD36 influences lipogenesis via SREBP1 processing [79], which is also elevated in lipopolysaccharide-induced M1 macrophages [80]. Additionally, miR-448 is overexpressed in VSMCs from atherosclerotic plaques compared with normal arteries [81]. Our study confirmed that both miR-335-5p and miR-448 target key inflammatory mediators, including NF-κB, IL-6, and TNF-α, linking them to CAS development. Multiple miRNAs—including miR-34a, miR-125a-5p, and miR-223—either directly target CD36 transcripts or modulate its downstream NF-κB/IL-6/RhoA pathway [82,83,84]. In this context, miRNA-targeting antisense oligonucleotides have entered mid-phase clinical trials; however, only a handful of miRNA-targeting drugs have undergone clinical testing so far, and none in the cardiovascular field. While the locked-nucleic-acid anti-miR-92a MRG-110 has completed first-in-human studies demonstrating potent, sustained target knockdown with an acceptable safety profile in healthy individuals [85], the anti-miR-132 CDR132L is being evaluated in the Phase II HF-REVERT study for post-MI heart failure [86], suggesting small interfering RNA drugs have already reached clinical reality. Evidence shows that VSMC hypercontraction results from increased Ca2+ sensitization via RhoA/ROCK activation, with inflammatory cytokines like TNF-α and IL-1β raising RhoA expression and activity [87]. In rat CAS models, NF-κB triggers the myosin light chain kinase/myosin light chain-2/endothelin-1 axis, further exacerbating VSMC contraction [26]. Taken together, these findings highlight miR-335-5p and miR-448 as regulators of Lp(a)/CD36-driven inflammation in CAS.

Amentoflavone, which is derived from traditional Chinese medicinal plants such as ginkgo biloba and *Selaginella tamariscina* in the treatment of cardiovascular diseases [88] and can now be produced by concise two-step dimerization of apigenin, is increasingly viewed as a drug-like small-molecule “mimetic” of genetic CD36 antagonist [89,90]. Oral gavage at 25–100 mg/kg/day for one week attenuates myocardial ischemia–reperfusion injury in rats, possibly via suppressing the NF-κB pathway [91], while the in vitro anti-pyroptotic effect of amentoflavone was demonstrated in attenuating the doxorubicin-induced cardiotoxicity inhibition of the stimulator of interferon gene/nod-like receptor protein 3 signaling pathway, thereby validating its efficacy as a cardioprotective agent [92]. The selection of 10 μM amentoflavone for functional assays in the present study was based on preliminary dose–response experiments (unpublished) showing that this concentration achieved maximal reversal of Lp(a)-induced proinflammatory responses without affecting baseline viability, consistent with the plateau seen in our viability curve between 10 μM and 25 μM. While detailed pharmacokinetic profiles of amentoflavone in the context of CAS are not yet available, previous in vivo studies have reported achievable plasma concentrations and tissue accumulation in the low μM range following amentoflavone administration in rodents [93,94,95]. While amentoflavone treatment elevated miR-335-5p and miR-448 levels above those in controls, this did not result in complete suppression of CD36 to below basal expression. As shown in Figure 5A,B and Figure 6E, CD36 mRNA and protein levels in the amentoflavone-treated group were reduced to levels comparable to controls, but not significantly lower. Therefore, the increase in miRNA expression likely reflects a compensatory overshoot in post-transcriptional regulation rather than supra-physiological silencing of CD36. Our findings revealed that amentoflavone mimicked shCD36 effects by attenuating Lp(a)-induced CD36 and RhoA-GTP expression, even in the presence of OE-CD36. It also epigenetically decreased CD36 expression and suppressed the downstream TNF-α/NF-κB/IL-6/RhoA-GTP/CD80 signaling via miR-335-5p and miR-448 in HCASMCs, suggesting that miRNA-based therapeutics hold great promise for treating inflammation-associated CAS.

Our study should be interpreted in light of several limitations. First, the relatively small sample size (n = 77) may limit statistical power and generalizability, and it precludes robust subgroup analyses (e.g., smoking status, sex stratification, medication use). Nonetheless, our preliminary exploratory evaluation suggested no obvious qualitative difference in the direction of association between smokers and non-smokers, although the statistical power was limited. Larger, adequately powered cohort studies are warranted to clarify whether smoking status modifies the Lp(a)/CD36 interaction in CAS. Second, baseline demographic and clinical differences—including a higher proportion of men in the CAS group, differences in and chronic β-blocker use, and other significant baseline parameters such as high-density lipoprotein cholesterol, hemoglobin, and monocyte counts—may influence baseline inflammatory and contractile pathways and thus confound associations despite our exploratory analyses. Due to the limited sample size, our regression models did not include multivariate adjustments; hence, the post hoc smoking stratification, while suggestive, was underpowered. Third, biological and technical variability in Lp(a) measurements, as well as unmeasured confounders (e.g., other lipid fractions, inflammatory mediators), may have influenced the results. Fourth, the study was conducted entirely in vitro; while molecular docking and mechanistic assays strongly support the role of the Lp(a)/CD36 axis, in vivo validation is needed. Fifth, the absence of oxidized low-density lipoprotein as a comparator limits the ability to fully distinguish Lp(a)-specific effects from those of other oxidized lipoproteins. Sixth, while we focused on delineating the mechanistic role of miR-335-5p and miR-448 in modulating Lp(a)/CD36 signaling within HCASMCs, which we validated through luciferase reporter assays and gain/loss-of-function experiments, quantifying their baseline circulating and cellular levels in CAS patients versus matched controls would add significant clinical relevance. Although such measurements were not included in the present work due to limited sample availability, recent clinical studies have shown that miR-335-5p and miR-448 are dysregulated in cardiovascular disease and vascular inflammation contexts, supporting their potential as CAS biomarkers. Owing to material and regulatory constraints, we were unable to perform patient-level miRNA measurements for this revision. The archived plasma/PBMC aliquots were exhausted by the prespecified assays (Lp(a), sCD36, flow cytometry), and residual material was not collected in RNA-stabilizing tubes, making de novo miRNA quantification unreliable. Additional sampling would require new consent and IRB amendment, which cannot be completed within the current decision window. To avoid over-interpretation, we have restricted all miRNA conclusions to mechanistic, in-vitro evidence and, therefore, their potential as biomarkers or therapeutic targets in CAS cannot be asserted. Seventh, Rho kinase activity was expressed as the pMBS/tMBS ratio and RhoA-GTP quantification, both of which are well-established surrogates of smooth muscle contractile activation in vascular biology. While these markers provide strong mechanistic evidence of RhoA-mediated signaling, direct functional assays—such as calcium sensitization measurements, myosin light chain phosphorylation, or collagen gel contraction assays—would more explicitly demonstrate HCASMC contractile responses. Due to the scope of the current work, these experiments were not performed; however, we propose them as a priority for future validation of the link between Lp(a)/CD36/RhoA activation and vascular smooth muscle hypercontractility in CAS. Eighth, while our workflow incorporated an initial rigid-body docking (ClusPro) followed by flexible refinement (HADDOCK) and 100 ns explicit-solvent molecular dynamics to partially account for conformational flexibility, we acknowledge that the use of AlphaFold and crystallographic ectodomain structures did not fully capture the dynamic nature of CD36 or Lp(a) in the lipid-rich plasma milieu. In particular, lipid interactions, membrane anchoring effects, and potential conformational rearrangements of CD36′s extracellular loops upon ligand binding were not explicitly modeled. Similarly, our current simulation omitted the oxidized phospholipid cargo of Lp(a), which may alter binding energetics and pose stability. These aspects could influence the interface geometry and binding kinetics in vivo. Future studies will incorporate full-length membrane-embedded CD36 models, Lp(a)-associated lipid components, and enhanced-sampling molecular dynamics or coarse-grained simulations to better mimic physiological conditions and capture lipid–protein cooperative effects. Finally, the cross-sectional design cannot establish temporality or causality between Lp(a)/CD36 activation and CAS onset.

## 4. Materials and Methods

### 4.1. Cells, Compounds, and Reagents

All PMDMs were cultured in the RPMI-1640 culture media (Sigma-Aldrich Corporation, St. Louis, MO, USA) supplemented with 10% fetal bovine serum (FBS), 2 mM glutamine, 50 µg/mL streptomycin, and 50 U/mL penicillin in 5% carbon dioxide humidified atmosphere incubator at 37 °C to 98–100% confluence. The HCASMCs (ATCC^®^ PCS-100-021™, American Type Culture Collection, Manassas, VA, USA) were cultured in smooth muscle cell growth medium 2 (#C-22062, PromoCell GmbH, Heidelberg, Germany). Cells were sub-cultured and media refreshed every 48 h. Human IL-6 (Sigma-Aldrich, Cat #407652, ≥95% purity, Lot #SLCL0481), LDL (Sigma-Aldrich, Cat #LP2, ≥95% purity, Lot #SLBP8776V), and amentoflavone (Sigma-Aldrich, HPLC grade ≥ 98%, Cat #A9034, Lot #MKBT9052V) were sourced from Sigma-Aldrich. Amentoflavone was prepared in DMSO (Sigma-Aldrich, Cat #D2650, Lot #BCBL1917V) and diluted in medium to obtain the concentrations for assays. Methylergonovine (Methergin^®^, Novartis, Basel, Switzerland, Batch #MTG-2109) and nitroglycerin (Millisrol^®^, G. Pohl-Boskamp, Hohenlockstedt, Germany, Lot #NTG-3459) were obtained from the respective manufacturers.

### 4.2. Study Population

This prospective cohort study was approved by the Taipei Medical University Joint Institutional Review Board (approval number: TMU-JIRB 201503007). All patients gave written informed consent for use of their blood in research, and the study followed the Declaration of Helsinki guidelines for biomedical research involving human subjects. This study enrolled 77 patients (35 men, 42 women) with chest pain and suspected ischemic heart disease on noninvasive tests, who underwent diagnostic coronary angiography (with or without CAS but no obstructive stenosis) from September 2019 to February 2021. The study participants were allocated to either the control group (n = 36) or the CAS group (n = 41). Of the participants, 4 individuals in the control group and 15 in the CAS group were identified as active smokers. CAS patient inclusion required spontaneous chest pain at rest with ST-segment changes on ECG relieved by sublingual nitroglycerin, no angiographic signs of obstructive CAD after intracoronary nitroglycerin, and a positive intracoronary methylergonovine provocation test. CAS was not induced in the remaining 36 patients (designated as the non-CAS control group), who exhibited atypical chest pain, lacked angiographic evidence of obstructive CAD, and demonstrated negative responses to intracoronary methylergonovine provocation testing. Atypical chest pain was defined as spontaneous or exertion-induced chest pain relieved by sublingual nitroglycerin without associated ST-segment changes on resting electrocardiogram [96]. Exclusion criteria were obstructive CAD, coronary microvascular spasm [97], inflammatory signs probably linked to noncardiac diseases (e.g., infections and autoimmune disorders), liver disease/renal failure (serum creatinine level > 2.5 mg/dL), collagen disease, malignancy, and loss of blood samples. No patients had allergies or hypersensitivities.

### 4.3. Patient Data Collection

Demographic, anthropometric, and laboratory data; comorbidity details; and medication use, habits, and functional unit counts were recorded for each patient. Current smoking was characterized as the consumption of at least 1 cigarette within the 3 weeks preceding cardiac catheterization. Diabetes mellitus was diagnosed if fasting glucose was ≥126 mg/dL on ≥2 occasions, or based on dietary or medical treatment. Baseline seated blood pressure was calculated as the average of 6 measurements obtained during the initial 2 office visits, which were scheduled 2 weeks apart. Hypertension was defined as blood pressure above 140/90 mmHg on ≥2 occasions or current antihypertensive therapy.

### 4.4. Spasm Provocation Test Protocol

Coronary angiography was performed using the Judkins method, with prior withdrawal of nitrates and calcium channel blockers for at least 24 h [98]. Obstructive CAD was defined as a ≥50% decrease in luminal diameter following intracoronary nitroglycerin [6]. If no obstructive CAD was found, intracoronary methylergonovine was administered stepwise (1, 5, 10, 30 μg) to the right coronary artery and then to the left. CAS was defined as a >70% reduction in luminal diameter compared with post-intracoronary nitroglycerin, accompanied by angina and/or ST depression or elevation [6]. Provocation testing was discontinued following an intracoronary injection of 50–200 μg of nitroglycerin.

### 4.5. Monocyte Isolation from Human Peripheral Blood

After overnight fasting prior to coronary angiography, blood was drawn into BD Vacutainer^®^ CPT™ tubes for mononuclear cell preparation (#362753, BD Diagnostics, Sparks Glencoe, MD, USA) and centrifuged at 1800× *g* at room temperature for 20 min. After discarding the upper plasma and Ficoll™Hypaque™(Amersham Biosciences, Uppsala, Sweden), and without disturbance of the red lowest layer, the opaque mononuclear cell interface was carefully transferred to a new 50 mL conical tube. The mononuclear cells were washed twice with phosphate buffered saline (PBS). Monocytes were then isolated using Invitrogen™ Dynabeads^®^ CD14 superparamagnetic beads (#11149D, Thermo Fisher Scientific Inc., Waltham, MA, USA) and magnetic activated cell sorting. Monocyte purity was measured by flow cytometry using fluorescein-labeled CD14-positive cells. Isolated monocytes were subsequently re-suspended in Invitrogen™ TRIzol™ reagent (#15596026, Thermo Fisher Scientific Inc., Waltham, MA, USA), and the total RNA extract was stored at −80 °C until use.

### 4.6. Monocyte Differentiation into Macrophages

Monocytes were incubated at 37 °C with 5% CO_2_ for 2 h to facilitate adherence. After removing nonadherent cells with the supernatant, adherent monocytes were washed with prewarmed 15 mL phosphate buffered saline (PBS), and the washing solution was aspirated. Subsequently, the ImmunoCult™-SF macrophage medium (#10961, STEMCELL Technologies Inc., Kent, WA, USA) was used for monocyte differentiation to macrophages [11].

### 4.7. HCASMCs Culture

HCASMC cells were obtained from Lonza (Lonza Walkersville, Walkersville, MD, USA; Cat #CC-2583, Lot #HC2938) and cultured in DMEM (Gibco, Cat #11965092, Lot #DMEM2244) supplemented with 10% FBS (Gibco, Grand Island, NY, USA; Cat #16000044, Lot #2297076). Cells were kept at 37 °C in 5% CO_2_, with medium changes every 3 days to ensure the cells remained healthy and active. For assays, cells in their 5th to 7th passages underwent 24 h serum starvation with 0.5% FBS, followed by stimulation using 20 ng/mL Platelet-Derived Growth Factor-BB (BioVision, Zürich, Switzerland; Cat #4577-50, Lot #PDGF1021) at 37 °C for 24 h, which readied the cells for the subsequent stages of our experimental analysis.

### 4.8. Lp(a) Assay

Native human Lp(a) was purchased from Cell Sciences (www.cellsciences.com; Newburyport, MA, USA; Cat. No. CRA169B). The supplier specifies a concentration of 1 mg mL^−1^ in PBS (no preservative), a buoyant density of 1.063–1.10 g mL^−1^, and ≥95% purity confirmed by SDS-PAGE; electrophoretic profiling showed negligible contamination with other plasma lipoproteins, with only a minor Apo-A1 band visible. The molecular-weight distribution of the preparation was 280–700 kDa and was quantified using a Human Lp(a) ELISA kit (Abcam, Cambridge, UK; Cat #ab212165, Lot #ELISA9083), with a detection limit of 17.2 ng/mL.

### 4.9. CD36 Expression Analysis in HCASMCs

To investigate the effects of Lp(a) on CD36-mediated signaling pathways, we employed both gene silencing and overexpression strategies. For knockdown, we utilized a commercially available short hairpin RNA targeting CD36 (Origene, Rockville, MD, USA; Cat # TL314090). For overexpression, a human CD36 plasmid (NM_001001548) with a tagged open reading frame and its corresponding empty vector were obtained from Origene (Cat #RC221976). HCASMCs were transfected with either the shCD36 construct or the CD36 overexpression (OE-CD36) plasmid using the Neon™ electroporation system (Invitrogen Neon Transfection System, Cat. No. MPK5000), achieving an approximate transfection efficiency of 80%. Scrambled shRNA and empty vector plasmids served as negative controls in the knockdown and overexpression experiments, respectively. To establish a stable OE-CD36 cell line, transfected HCASMCs were subjected to selection with puromycin (5 μg/mL). The efficacy of CD36 knockdown and overexpression was confirmed by Western blot analysis.

### 4.10. RNA Processing and Quantitative Reverse Transcription Polymerase Chain Reaction (qRT-PCR) for CD36, RhoA, and miRNA

Total RNA was extracted using a guanidinium isothiocyanate buffer followed by one step of phenol-chloroform-isoamyl alcohol extraction. For reverse transcription, 1 μg of this RNA was incubated with 200 U of reverse transcriptase in a 20 μL reaction buffer. This buffer included 0.25 μg of random primers and 0.8 mM dNTPs, and the incubation occurred at 42 °C for 1 h. The resulting first-strand cDNA served as the template for the PCR; thereafter, we used 20 ng aliquots of cDNA in each 20 μL reaction mix containing 10 μM primer, 2× Real Quantitative PCR Master Mix, and 6 μL DEPC H_2_O. The PCR was conducted using the SYBR Green method on a MxPro-Mx3000P Quantitative PCR machine (Stratagene, La Jolla, CA, USA). Each sample underwent triplicate runs, with GAPDH as the reference gene. For miRNA quantification, total RNA from both cells and tissue samples was extracted using Trizol reagents (Invitrogen, Life Technologies, Carlsbad, CA, USA). We then synthesized the first-strand cDNA using the PrimeScript RT Reagents Kit (Takara, San Jose, CA, USA). The miRNA levels in the cells were quantified using qRT-PCR, with U6 miRNA as the normalization control. This qRT-PCR was performed using an SYBR Premix Ex Taq Kit (Takara) on a QIAGEN rotor real-time PCR machine (QIAGEN, Valencia, CA, USA).

### 4.11. Molecular Docking

The soluble ectodomain of CD36 (AlphaFold model AF-Q07954-F1, residues 30–439, Protein Data Bank, downloaded from https://alphafold.ebi.ac.uk/) and apolipoprotein(a) crystal structure Protein Data Bank file downloaded from https://www.rcsb.org/structure/1AV1 (accessed on 12 June 2025) were trimmed, protonated at pH 7.4, and energy-minimized with AMBER ff19SB. Rigid-body sampling was carried out on the ClusPro 2.0 server (balanced energy set-up; ~70,000 fast Fourier transform rotations, 1 Å grid). The 1000 best poses were clustered at 9 Å Cα Root-Mean-Square Deviation and ranked by cluster size; the 10 highest-scoring centers (docking scores −273.47 to −225.09 kcal mol^−1^; confidence 0.82–0.92) were forwarded to HADDOCK 2.4 (Guru mode, web server http://hdock.phys.hust.edu.cn). In HADDOCK, Lys70/Lys74 of apolipoprotein(a) and Asp238/Glu240/Glu242 of CD36 were defined as active residues; default passive residues were automatically assigned. Each run produced 2000 rigid-body (it0), 200 semi-flexible (it1), and 20 water-refined models, followed by fraction of common contacts clustering at 1.0 Å. The top cluster (128 models) yielded an average HADDOCK score of −122 ± 8 kcal mol^−1^ and buried ~1600 Å^2^ of interface surface, defining the final docked complex used for subsequent analyses.

### 4.12. Western Blot Analysis

Western blotting was performed according to standard protocol [99] using antibodies against CD36 (Cell Signaling Technology, Danvers, MA, USA; Cat #74002, Lot #CD362031), IL-6 (Cell Signaling Technology, Cat #12153, Lot #IL61213), TNF-α (Cell Signaling Technology, Cat #3707, Lot #TNF7081), NF-κB (Cell Signaling Technology, Cat #8242, Lot #NFkB1093), CD80 (Abcam, Cat #ab134120, Lot #CD801209), GAPDH (Abcam, Cat #ab9484, Lot #GAP2203), RhoA (Cell Signaling Technology, Cat #2117), RhoA-GTP (Cat #8820), ROCK1 (Cat #4035), ROCK2 (Cat #9029), t-MBS (Cat #2634), and p-MBS (Cat #3040). Protein bands were detected using the Amersham ECL system (GE Healthcare, Piscataway, NJ, USA) and quantified via ImageJ software, version 1.54k, National Institutes of Health, Bethesda, MD, USA (https://imagej.nih.gov/ij/).

### 4.13. Flow Cytometry Analysis

Approximately 2 million cells were fixed using 4% formaldehyde (Sigma-Aldrich, Cat #F8775, pH 7.5, Lot #FA9081) for 15 min at ambient temperature. Cells were then incubated in a blocking buffer containing 1% bovine serum albumin (Sigma-Aldrich, Cat #A7906, Lot #BSA2023) and 1% goat serum (Gibco, Cat #16210072, Lot #GS12345) in PBS for 30 min. Cells were then treated for 2 h with primary antibodies targeting CD36 (Abcam, Cat #ab133625, Lot #CD36988) and RhoA (Abcam, Cat #ab54835, Lot #RHA6521), each diluted 1:100. After washing, cells were incubated for 1 h in PBS containing fluorescein isothiocyanate-conjugated IgG secondary antibody (Sigma-Aldrich, Cat #F6257, Lot #FITC7742). Surface marker expression was quantified using a BD fluorescence-activated cell sorting Calibur flow cytometer (BD Biosciences, San Jose, CA, USA). For PMDM analysis, events were gated by FSC/SSC to exclude debris, followed by singlet discrimination (FSC-A vs. FSC-H), viability gating, and final identification of the CD36^+^ population. Approximately 2 × 10^6^ cells were fixed in 4% formaldehyde (Sigma-Aldrich, Cat. F8775; pH 7.5; Lot FA9081) for 15 min at room temperature. Cells were then blocked for 30 min in PBS containing 1% bovine serum albumin (Sigma-Aldrich, Cat. A7906; Lot BSA2023) and 1% goat serum (Gibco, Cat. 16210072; Lot GS12345). Primary antibodies against CD36 (Abcam, Cat. ab133625; Lot CD36988) and RhoA (Abcam, Cat. ab54835; Lot RHA6521) were applied at 1:100 for 2 h. After washing, cells were incubated for 1 h with an FITC-conjugated IgG secondary antibody (Sigma-Aldrich, Cat. F6257; Lot FITC7742) in PBS. Surface marker expression was quantified on a BD FACSCalibur flow cytometer (BD Biosciences, San Jose, CA, USA).

### 4.14. Cell Viability Assessment After Amentoflavone Treatment

Cell viability following amentoflavone treatment was assessed using the MTT (3-(4,5-dimethylthiazol-2-yl)-2,5-diphenyltetrazolium bromide) assay. HCASMCs and CAS PMDMs were incubated with increasing concentrations of amentoflavone (12.5–100 μM) for 24 h, after which MTT reagent (0.5 mg/mL) was added for 4 h at 37 °C. Formazan crystals were dissolved in DMSO, and absorbance was measured at 570 nm using a microplate reader. Viability was expressed as a percentage relative to untreated controls.

### 4.15. Statistical Analysis

All assays were conducted in triplicate at least three times. Continuous variables were reported as mean ± standard deviation or median (interquartile range). Variables with positive skewness were log-transformed prior to conducting intergroup Student’s *t*-tests. Discrete variables were presented as counts and percentages of the total sample, with group comparisons conducted via the two-tailed Fisher’s exact test. Categorical variables were evaluated using the χ^2^ test. The Spearman correlation was used to assess the relationship between serum Lp(a) levels and sCD36 expression in patients with CAS. Linear regression was used to compute the coefficient of determination (r^2^) and its *p*-value. All statistical analyses were performed with SPSS (IBM Corp. Released 2017. IBM SPSS Statistics for Windows, Version 25.0 Armonk, NY, USA: IBM Corp.). A two-tailed *p*-value < 0.05 was regarded as statistically significant.

## 5. Conclusions

In CAS, higher serum Lp(a) levels were positively correlated with the levels of sCD36, which was not observed in controls. Lp(a) activated macrophage M1 polarization and increased RhoA and CD36 expression in PMDMs of CAS patients and HCASMCs, resulting in elevated sCD36 protein in PMDMs. In Lp(a)-treated HCASMCs, shCD36 significantly decreased both protein and mRNA expression levels of CD36, RhoA-GTP, RhoA, IL-6, TNF-α, NF-κB, and CD80. In addition, shCD36 and amentoflavone significantly reduced the RhoA-GTP/RhoA ratio. Conversely, OE-CD36+Lp(a) induced a significantly higher RhoA-GTP/RhoA ratio than Lp(a) alone. The addition of amentoflavone to OE-CD36 significantly downregulated the CD36 expression level to the extent similar to that with shCD36. Amentoflavone mediated Lp(a)/CD36 interaction in HCASMCs through epigenetic regulation of CD36, IL-6, TNF-α, and NF-κB mRNA expression via miR-335-5p and miR-448. Our study provides a novel Lp(a)-triggered CD36-dependent TNF-α/NF-κB/IL-6/RhoA-GTP signaling pathway mediating HCASMC dysfunction in CAS (see Graphical Abstract), which makes CD36 and its downstream effectors promising therapeutic targets.

## Figures and Tables

**Table 1:** Baseline characteristics of the study cohort.

	Controls (n = 36)		CAS (n = 41)		*p*-Value	
Age, years	56.6 ± 15.4		56.3 ± 11.9		0.94	
Male sex, n (%)	12 (33)		23 (56)		0.045	
Body mass index, kg/m^2^	24.5 ± 4.1		26.0 ± 4.5		0.12	
Current smoker, n (%)	4 (11)		15 (41)		0.004	
Diabetes mellitus, n (%)	4 (11)		2 (5)		0.38	
Hypertension, n (%)	5 (14)		9 (24)		0.26	
Systolic blood pressure, mmHg	113 ± 14		115 ± 17		0.56	
Diastolic blood pressure, mmHg	67 ± 10		71 ± 10		0.10	
Heart rate, beats/min	67 ± 9		71 ± 14		0.24	
Left ventricular ejection fraction, %	65 ± 5		65 ± 7		0.98	
Total cholesterol, mg/dL	171 ± 35		166 ± 33		0.56	
Triglyceride, mg/dL	85 ± 51		99 ± 54		0.26	
HDL cholesterol, mg/dL	53 ± 12		47 ± 12		0.034	
LDL cholesterol, mg/dL	95 ± 29		98 ± 27		0.66	
Lipoprotein(a), mg/dL	18.6 ± 8.7		75.9 ± 29.4		0.001	
sCD36	2.1 ± 1		6.6 ± 3.3		0.001	
Peripheral leukocytes, /mm^3^	6006 ± 1459		6786 ± 2182		0.088	
Monocytes, /mm^3^	458 ± 159		551 ± 178		0.027	
Macrophage, /mm^3^	113 ± 36		411 ± 113		0.001	
Lymphocytes, /mm^3^	1725 ± 643		1669 ± 866		0.76	
Hemoglobin, g/dL	13.4 ± 1.5		14.1 ± 1.3		0.03	
Hematocrit, %	39.0 ± 5.0		41.3 ± 3.6		0.026	
Platelets, ×10^3^/mm^3^	222 ± 57		241 ± 61		0.18	
hs-CRP, mg/L *	0.63 (0.26–1.40)		0.70 (0.21–0.98)		0.31	
Provoked coronary artery						
Left anterior descending artery, n (%)			10 (24)			
Left circumflex artery, n (%)			1 (2)			
Right coronary artery, n (%)			32 (78)			
Number of spastic arteries						
One-vessel spasm, n (%)			35 (90)			
Two-vessel spasm, n (%)			4 (10)			
Three-vessel spasm, n (%)			0 (0)			
Medications	A	D	A	D	A	D
Aspirin, n (%)	31 (86)	33 (81)	31 (89)	36 (88)	0.51	0.92
β-blockers, n (%)	34 (94)	34 (83)	18 (51)	7 (17)	0.12	0.001
Calcium channel blockers, n (%)	4 (11)	11 (27)	17 (49)	39 (95)	0.08	<0.001
Diuretics, n (%)	0 (0)	1 (2)	1 (3)	1 (2)	0.35	0.91
Angiotensin receptor blockers, n (%)	7 (19)	12 (29)	7 (20)	11 (27)	0.32	0.49
Nitrates, n (%)	1 (3)	1 (2)	1 (3)	0 (0)	0.93	0.28
Statins, n (%)	10 (28)	20 (49)	11 (31)	22 (54)	0.06	0.05

Values are presented as mean ± standard deviation or median (interquartile range). A, before angiography; CAS, coronary artery spasm; D, at discharge; hs-CRP, high-sensitivity C-reactive protein; HDL, high-density lipoprotein; LDL, low-density lipoprotein. * Log-transformed values were performed prior to analysis.

## Data Availability

Data are contained within the article and the Supplementary Materials. Further inquiries can be directed to the corresponding author.

## Supplementary Materials

The following supporting information can be downloaded at: https://www.mdpi.com/article/10.3390/ph18091384/s1, Supplementary Figure S1: qRT-PCR analysis of canonical M1/M2 macrophage markers in CAS PMDMs following Lp(a) treatment. PMDMs were exposed to 1 µM Lp(a) for 24 h, and relative mRNA expression levels of inducible nitric oxide synthase (iNOS) and interleukin-12 (IL-12) (M1 markers), as well as arginase-1 and interleukin-10 (IL-10) (M2 markers), were quantified by qRT-PCR. Supplementary Figure S2: (A) We used miRTarBase and MirTarget to identify miRNAs targeting CD36, presented as a radial connectivity chart of CD36 and candidate miRNAs. (B) Amentoflavone upregulated miR-335-5p and miR-448, while it downregulated miR-455-5p and miR-155-5p. Supplementary Figure S3: A, B, C, D represent full-size Western blots of Figure 5A, Figure 5C, Figure 5D, and Figure 5E, respectively.
