# Supplementary material for: Lipoprotein(a)/CD36 Interaction Drives IL-6/RhoA-GTP Signaling and miRNA Epigenetic Regulation in Coronary Artery Spasm
Source: Pharmaceuticals (Basel). 2025 Sep 16;18(9):1384. doi: 10.3390/ph18091384 (PMC12472529; doi:10.3390/ph18091384)
Supplement: Supplementary file 1 [file pharmaceuticals-18-01384-s001.zip › pharmaceuticals-3770305-supplementary.pdf]

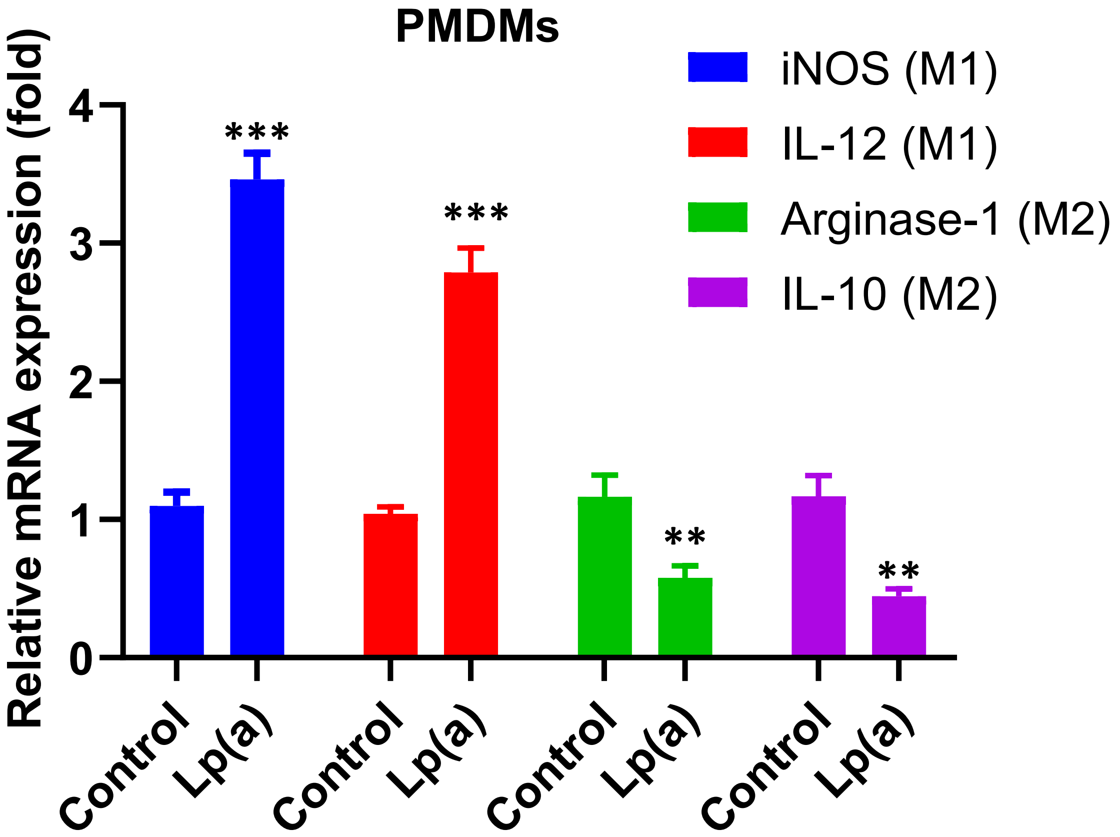

Supplementary Figure S1. (**A**) qRT-PCR analysis of canonical M1/M2 macrophage markers in CAS PMDMs following Lp(a) treatment. PMDMs were exposed to 1 μM Lp(a) for 24 h, and relative mRNA expression levels of inducible nitric oxide synthase (iNOS) and interleukin-12 (IL-12) (M1 markers), as well as arginase-1 and interleukin-10 (IL-10) (M2 markers), were quantified by qRT-PCR. Data are presented as fold change relative to untreated controls, normalized to GAPDH expression. Lp(a) significantly increased M1 marker expression while decreasing M2 marker expression, consistent with a shift toward a proinflammatory M1 phenotype. Error bars represent standard deviation (SD) from three independent experiments. $p < 0.05$ vs. control. **$p < 0.01$, ***$p < 0.001$.

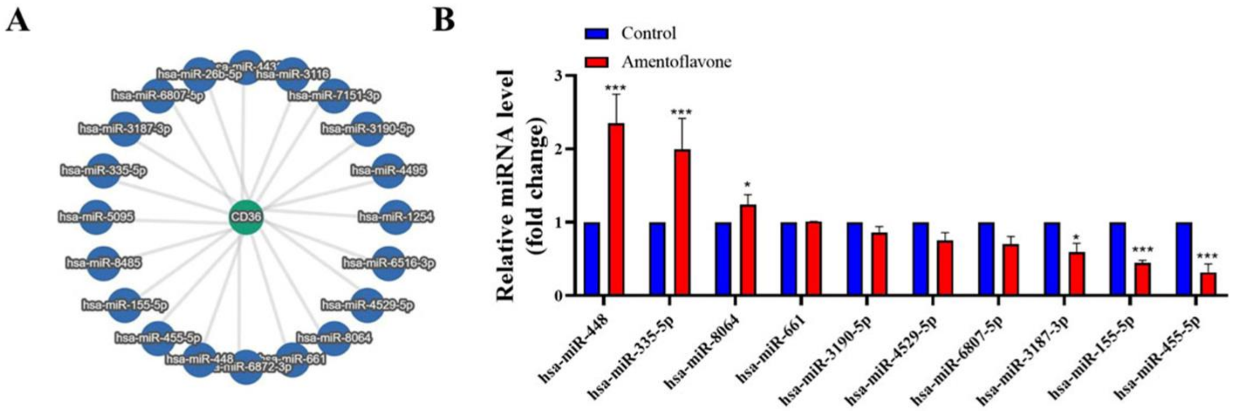

Supplementary Figure S2. (**A**) We used miRTarBase and MirTarget to identify miRNAs targeting CD36, presented as a radial connectivity chart of CD36 and candidate miRNAs. (**B**) Amentoflavone upregulated miR-335-5p and miR-448, while downregulated miR-455-5p and miR-155-5p. *p < 0.05, **p < 0.01, ***p < 0.001.

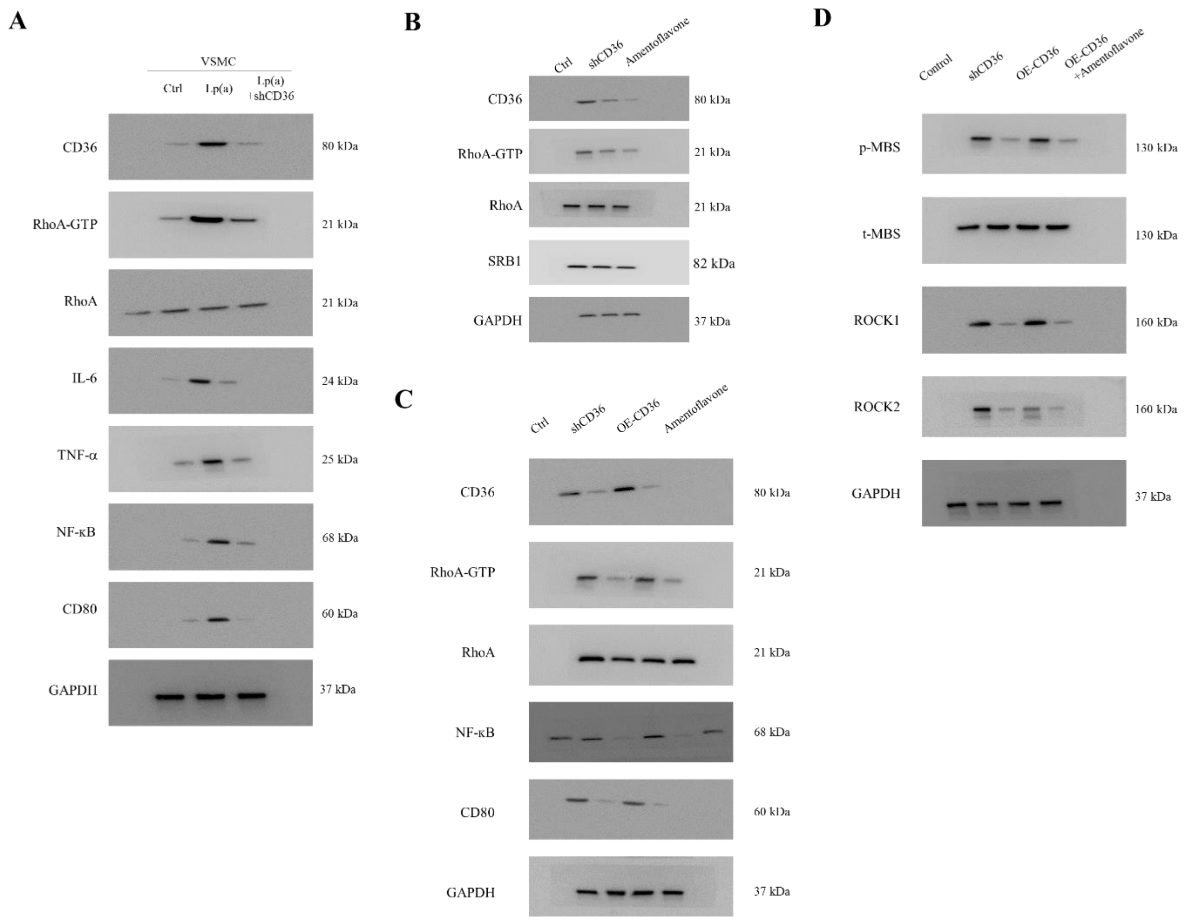

Supplementary Figure S3. A, **B**, **C** and **D** represent full-size western blots of Figure **5A**, **5C**, **5D** and **5E** in the Results section of the main text.
